# Development of a multiplex assay based on chimeric citrullinated peptides as proof of concept for diagnosis of rheumatoid arthritis

**DOI:** 10.1371/journal.pone.0215927

**Published:** 2019-05-02

**Authors:** Cristina García-Moreno, María José Gómara, María José Bleda, Raimon Sanmartí, Isabel Haro

**Affiliations:** 1 Unit of Synthesis and Biomedical Applications of Peptides, Institute of Advanced Chemistry of Catalonia (IQAC-CSIC), Barcelona, Spain; 2 Arthritis Unit, Hospital Clinic, Barcelona, Spain; Duke University School of Medicine, UNITED STATES

## Abstract

Anti-citrullinated peptide/protein antibodies (ACPAs) are the most specific serological biomarkers for rheumatoid arthritis (RA). They have both diagnostic and prognostic value, and are related to more aggressive joint disease in RA. However, a single biomarker cannot differentiate RA subtypes. So, simultaneous analysis of target citrullinated peptides, using a multiplex array based on chimeric peptides composed of several domains of human proteins, could be useful. In this work, eight chimeric peptides and the corresponding native arginine-containing control peptides were obtained by solid-phase peptide synthesis. The study included RA and psoriatic arthritis (PsA) patients attending the Rheumatology Unit of the Hospital Clinic in Barcelona, as well as healthy blood donors (BD) at the same hospital. Our main aim was to explore the diagnostic value of the novel multiplex array compared to a commercial ELISA-based ACPA assay in a serum-saving way. Using the combination of the eight chimeric peptide antigens in the multiplex array, 61.4% of the RA cohort were positive for 3 or more peptides; while, the healthy BD and PsA cohorts did not show any reactivity with the tested peptides. These results indicate that we have developed a highly specific multiplex assay based of chimeric citrullinated peptides derived from filaggrin, fibrin, vimentin and human enolase proteins for the detection of ACPAs in a serum-saving way.

## Introduction

Rheumatoid arthritis (RA) is a chronic and incapacitating inflammatory disease of the joints which is estimated to affect 0.5%-1% of the population worldwide. Long-lasting and the more severe cases can also develop into a systemic disease and have extra-articular effects [1,2]. As symptoms do not always appear in the early stages of the disease, there is a clear need to improve both the precision of specific tests for its diagnosis, and its early differentiation from other rheumatic diseases that affect the articulations and connective tissue. This is especially so in the case of patients with a poor prognosis or those in the early developmental stages of the disease.

In recent years, several posttranslational modifications have been reported in the context of RA, such as citrullination and carbamylation of proteins as well as proteins containing MAA (malondialdehyde-acetaldehyde) adducts which co-localize in the inflamed synovial tissue of RA patients [3,4]. However, the highest specificity for the diagnosis of RA is achieved via analysis of deiminated peptides or proteins, i.e., epitopes containing citrulline residues [5]. Anti-citrullinated peptide/protein antibodies (ACPAs) are the most specific serological biomarkers for RA. They have both diagnostic and prognostic value, and are related to a more aggressive joint disease in RA. However, a single biomarker cannot differentiate RA subtypes, so simultaneous analysis of the target citrullinated peptides, incorporated into a multiplex test, would facilitate the biological fingerprinting of autoantibodies in serum that would allow us to identify subgroups of patients with specific clinical characteristics. These may include different prognoses; and those who either respond well to, or suffer negative effects from, certain therapeutic interventions.

Within this context, in previous work we already mapped the epitope anti-citrullinated fibrin and vimentin antibody responses using synthetic peptides obtained by solid-phase peptide synthesis, with the aim of improving the balance between sensitivity and specificity. The peptides selected were covalently combined to render several chimeric peptides bearing fibrin, vimentin and filaggrin domains [6,7]. We have demonstrated that the presence of different peptide sequences within the same molecule can result in synergistic effects compared to the monomeric peptides or the corresponding physical mixture of them [6,8,9]. Our previous results have shown that there are potential applications of RA diagnostic systems based on chimeric peptides composed of several citrullinated domains of human proteins. Moreover, those findings imply that more than one serological test that combine these target peptides in a single analysis is required to classify patients based on the presence or absence of ACPAs.

In this work, we now aim to analyze eight chimeric citrullinated peptides derived from different proteins present in rheumatoid synovial fluid simultaneously, incorporating them into a multiplex test. This will thereby allow us to explore the diagnostic value of the multiplex array compared to an ELISA-based commercial ACPA assay in a serum-saving way. These results could have far-reaching practical implications in the future for establishing whether the multiplex system adds power to current RA tests.

## Materials and methods

The work was approved by the Ethics Committees of the *Consejo Superior de Investigaciones Científicas* (CSIC), Madrid, Spain, and of the Hospital Clinic, Barcelona, Spain. All methods were performed in accordance with the relevant IQAC-CSIC guidelines and regulations. Written consent was obtained from all participants and suitably informed.

### Chimeric peptides

**Fig 1:**
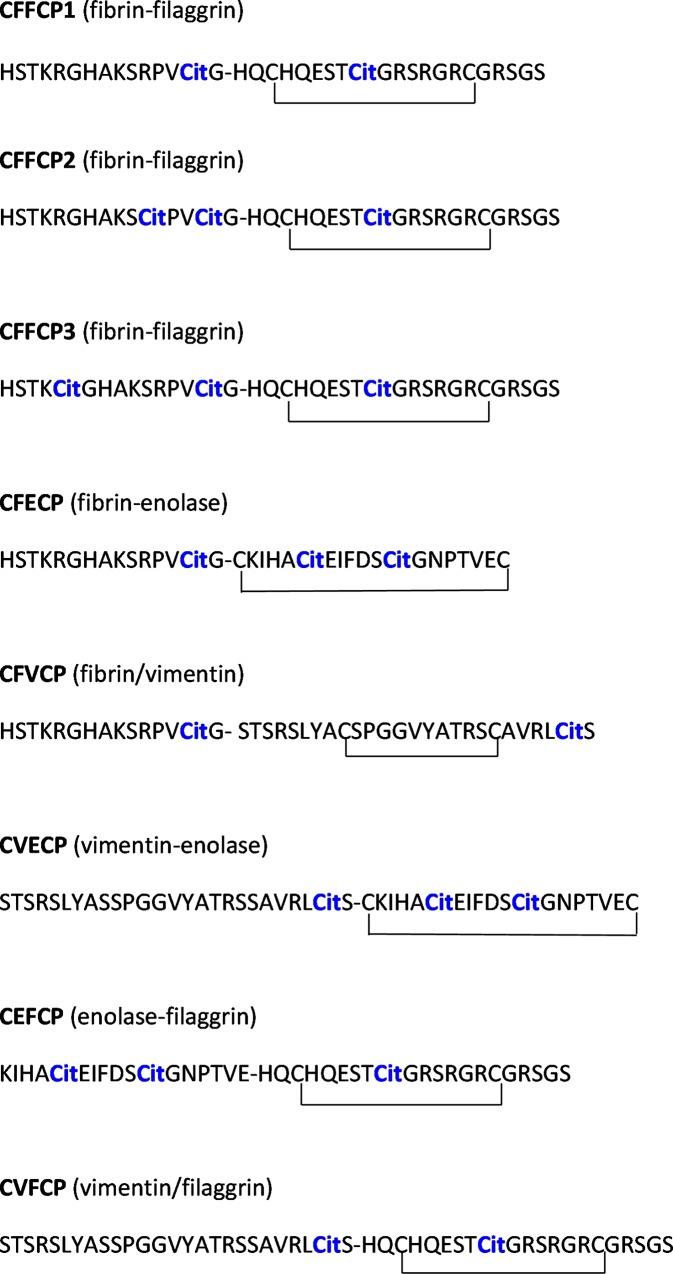
Primary structure of chimeric citrullinated peptides derived from fibrin, filaggrin, vimentin and enolase proteins.

The eight chimeric peptides shown in Fig 1 were manually synthesized as C-terminal carboxamides in polypropylene syringes, each fitted with a polyethylene porous disk. 9-fluorenyl-methoxycarbonyl (Fmoc)-protected amino acids (3 equiv) and 2-(1H-7-azabenzotriazole-1-yl)-1,1,3,3-tetramethyluronium hexafluorophosphate methanaminium (HATU) (3 equiv) were added sequentially to the resin in dimethylformamide (DMF) (3 mL) followed by diisopropylethylamine (DIPEA) (6 equiv).

The mixture was allowed to react, with intermittent manual stirring, for 30 min. The solvent was removed by filtration, and the resin was washed with DMF (5 × 30 s). The extent of coupling was checked by applying the Kaiser colorimetric assay or the De Clercq test. The Fmoc group was removed by treating the resin with 20% piperidine in DMF (3–4 mL/g resin, 2 × 10 min). Peptide elongation continued by coupling the second and subsequent amino acids using the same procedure. The peptides were cleaved from the resin by means of treatment with 95% trifluroacetic acid (TFA) in the presence of scavengers: 2% H_2_O, 2% 1,2-ethanedithiol (EDT) and 1% triisopropylsilane (TIS) for 3 h. In order to synthesize the cyclic chimeric peptide, 2 serine residues in the linear peptide were substituted for 2 cysteines. After peptide cleavage, the peptide was dissolved in acetic acid (AcOH)/H_2_O (1:1, 3 mg/mL) under N_2_, then HCl (1 M, 0.1 mL/mg) followed by I_2_ (20 equiv/acetamidomethyl, Acm) were added to the solution. After 4 h, the I_2_ was quenched by adding 1 M ascorbic acid drop-wise until the mixture became colorless and then it was concentrated by evaporation under reduced pressure to approximately one third of the original volume. The final product was purified by semipreparative HPLC in a Kromasil C-18 column (Tecknokroma, 5 μm, 25 x 1 cm) with a linear gradient of 100%-75% A in B over 30 min, at a flow rate of 4 mL/min, using 0.05% TFA in water (A) and 0.05% TFA in acetonitrile (B) as the eluting system. The purified chimeric peptides (purity greater than 95%) were characterized by analytical ultra-performance liquid chromatography (UPLC) and electrospray ionization mass spectrometry (ES-MS).

### Serum specimens

Patients who fulfilled revised ACR/EULAR 2010 criteria for the classification of RA were enrolled in this study. They were all outpatients attending the Rheumatology Unit of the Hospital Clinic in Barcelona. Serum samples used as negative controls were obtained from blood donors at the same hospital. Sera were previously tested in duplicate for the presence of anti-CCP3 (Immunoscan RA; Eurodiagnostica, distributed by Diasorin, Madrid, Spain) and anti-CFFCPs, anti-CVFCP and anti-CFVcCP as previously described [6, 7]. Patients with psoriatic arthritis (PsA) meeting CASPAR criteria [10] were included as an inflammatory control group.

### ELISA assays

Peptide sequences were coupled covalently to ELISA microplates (Costar Corp., DNA-bind N-oxysuccinimide surface, Cambridge, MA, USA) as previously described [6, 7]. Briefly, the peptides were diluted to 10 μg/mL in 0.05 M carbonate/bicarbonate buffer (pH 9.6). Then, 100 μL of the peptide solution was added to each well of microplates and incubated overnight at 4°C. Each plate contained control wells that included all reagents except the serum sample in order to estimate the background reading, together with control wells that included all reagents except the peptide to evaluate non-specific serum reactions. For blank controls, wells were coupled with 2 μg BSA/well. After incubation, the plates were blocked with 2% bovine serum albumin (BSA) in 0.05 M carbonate/bicarbonate buffer (pH 9.6) for 1 h at room temperature. Sera were diluted 50-fold in RIA buffer (1% BSA, 350 mM NaCl, 10 mM Tris-HCl, pH 7.6, 1% vol/vol Triton X-100, 0.5% wt/vol Na-deoxycholate, 0.1% SDS) supplemented with 10% fetal bovine serum. Afterwards, 100 μL/well was added and incubated for 1.5 h at room temperature. After washing 6 times with phosphate-buffered saline (PBS)/0.05% Tween-20, we added 100 μL/well of anti-human IgG conjugated to peroxidase diluted 1:1000 in RIA buffer. After incubation for 1 h at room temperature, the plates were washed 6 times with PBS/0.05% Tween-20 and bound antibodies were detected with o-phenylenediamine dihydrochloride (OPD, Sigma-Aldrich, St. Louis, MO, USA and 0.8 μL/mL 30% hydrogen peroxide. The plates were incubated at room temperature for 30 min. The reaction was stopped with 50 μL of 2N H_2_SO_4_ per well and absorbance values were measured at a wavelength of 492 nm. All the sera were tested in duplicate. Control sera were also included to monitor inter- and intra-assay variations.

### Microarray design

To perform microarray assays, the peptides were covalently immobilized (Miniarrayer BioOdisseyTM CalligrapherTM, BioRad, CA, USA) on the slides via epoxide groups. The immunoassay procedure was carried out as described previously [11]. A secondary antibody anti-human IgG labeled with Alexa Fluor 647 was used and the fluorescence of the spots was quantified in a scanner (ScanArray GX Plus, Perkin Elmer). The microarray assay was then optimized in two steps.

Firstly, the plates were blocked with 2% BSA in 0.05 M carbonate/bicarbonate buffer (pH 9.6) for 1 h at room temperature. Afterwards, serum samples (diluted 1:50) were analyzed for the presence of ACPA-specific IgG working with three chimeric peptides (CFFCP1, CFFCP2 and CFFCP3) that were spotted in quintuplicate on: 1) glass slides coated with an epoxy group or 2) Super NHS microarray substrate slides (ArrayIt, CA, USA). After 1.5 h of incubation at room temperature in a humidity chamber, the slides were washed six times with PBS tween buffer and the anti-human IgG labeled with Alexa Fluor 647 (dilution 1:250) was added and incubated at room temperature for 1 h. Microarray fluorescence was read using excitation and emission wavelengths of 633 and 647 nm, respectively. The Scan Array GX Plus software calculates intensity homogeneity (as a coefficient of within individual spots and intra-spot variabilities) for all spots coated with the same peptide. In this first assay, the glass slides coated with epoxy groups yielded better results.

Secondly, the same procedure as indicated above was carried out but diluting the peptides in two types of buffer: 1) carbonate buffer pH 9.6 or 2) peptide printing buffer (ArrayIt, CA, USA). The three peptides were spotted in quintuplicate on glass slides coated with an epoxy group using 18 and 6 randomly selected RA and 6 BD samples respectively (24 was the maximum number of samples on a slide). Microarray slides spotted with peptides diluted in carbonate buffer showed better results than those in the commercial buffer. Consequently, the final microarray assay that we performed with all the chimeric peptides was carried out in this way. Nevertheless, in order to improve spot intensity, the peptides/serum incubation time was increased to 2 h and also a lower dilution of anti-human IgG labeled with Alexa Fluor 647 (1:100) was used. The eight chimeric peptides were spotted in triplicate in the final assay. At least two of the three spots should be accepted to obtain results. After this, sera that still rendered a high coefficient of variation (CV) were discarded.

### Statistical analysis

Cut-off values for homemade ELISA tests were selected according to receiver operating characteristic (ROC) analysis, to give a specificity of 98% for RA versus healthy blood donors (BD). In order to evaluate the diagnostic properties of the different peptides, sensitivity, specificity and positive and negative predictive values (PPV and NPV) were calculated using the RA and BD cohorts. Quantitative data were reported as means, standard deviations, medians and interquartile ranges; absolute frequencies and percentages were used to describe qualitative data.

The association between microarray and ELISA values was analyzed using the nonparametric Spearman rank correlation coefficient due to the nonsymmetric distributions of the data. To compare percentages, the classical two-sample test of proportions was used. All p-values were two sided and the significance level was established at α = 0.05. Comparison and analysis of ROC curves and all other statistical analysis was performed using STATA 15.0 statistical software [12].

## Results and discussion

**Fig 2:**
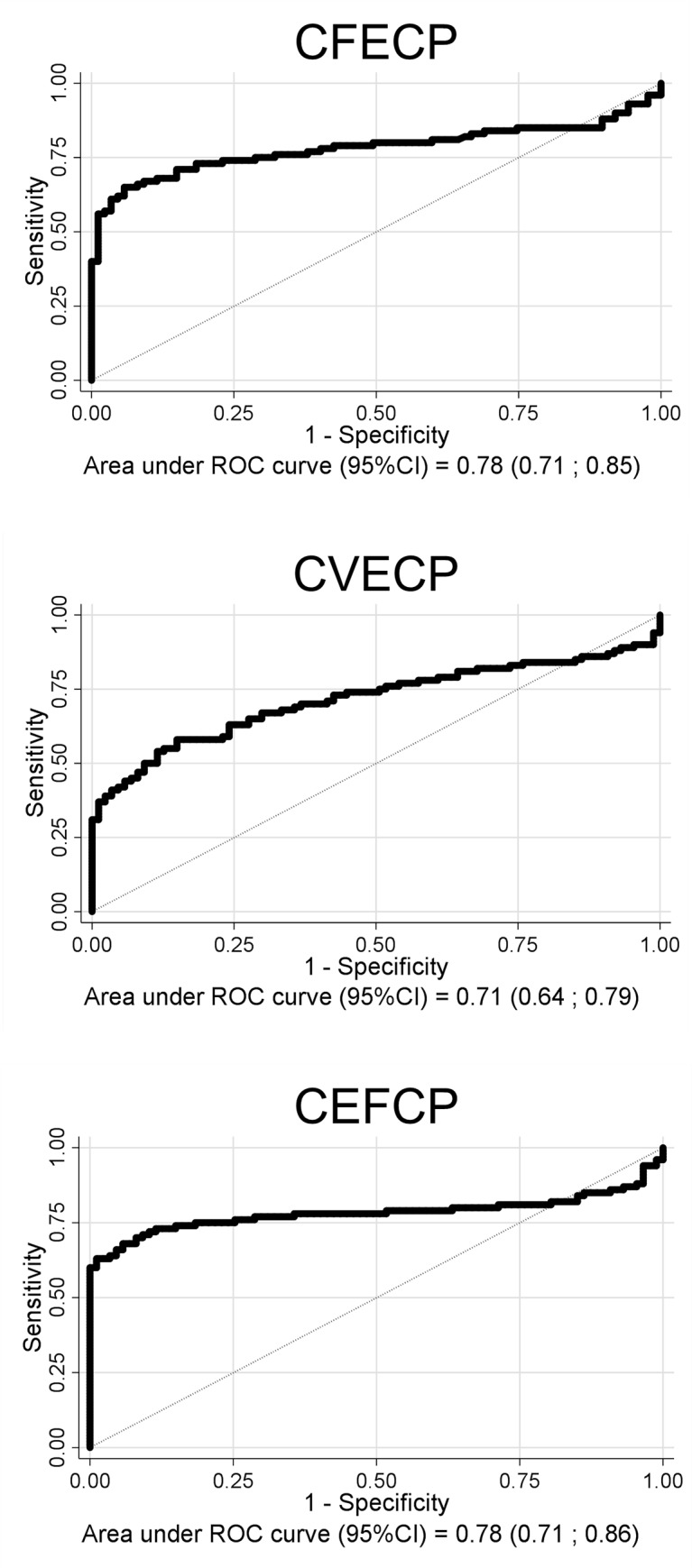
ROC curve analysis from ELISA results with chimeric fibrin-filaggrin-vimentin-enolase citrullinated peptides (CEFCP, CVECP and CFECP) in the cohort of patients with RA (n = 100) and of blood donors (n = 87).

As previously reported [6,7], the identification of novel citrullinated antigens that could supplement or complement ACPA-based existing tests, is required. A challenging approach consists of analyzing ACPA profiles using multiple citrullinated peptide antigens simultaneously. In the present work, considering the increased antigenicity previously observed with multimeric peptides bearing different epitope peptide sequences within the same molecule, we designed and synthesized three new chimeric peptides in the solid phase. They were composed of citrullinated peptides derived from filaggrin (cyclic filaggrin peptide, constituting the CCP1 test), fibrin (617–631), vimentin (47–72) and enolase (CEP-1), and herein we refer to them as chimeric fibrin/enolase citrullinated peptide (CFECP), chimeric vimentin/enolase citrullinated peptide (CVECP) and chimeric enolase/filaggrin citrullinated peptide (CEFCP). The analytical characterization of these three novel chimeric peptides using UPLC-MS is shown in the Supplementary material (Figs A, B and C in S1 File) and the primary structure of all the chimeric citrullinated peptides studied in the present work is shown in Fig 1. Firstly, a comparative ELISA assay was performed in cohorts of 100 RA and 87 BD sera using CFECP, CVECP and CEFCP as coating antigens; as was previously performed with the chimeric peptides CFFCP1, CFFCP2, CFFCP3, CFVCP and CVFCP [6, 7]. The values of the area under the ROC curve (RA vs BD) are shown in Fig 2: CEFCP: 0.78; CVECP: 0.71; CFECP: 0.78. Using cut-off values that yield a specificity of 98.85% with respect to BD, the sensitivity of the ELISAs was 63% for CEFCP, 37% for CVECP, and 56% for CFECP; with PPV > 97.4% in all three tests and NPV between 57.7% and 69.9%.

**Fig 3:**
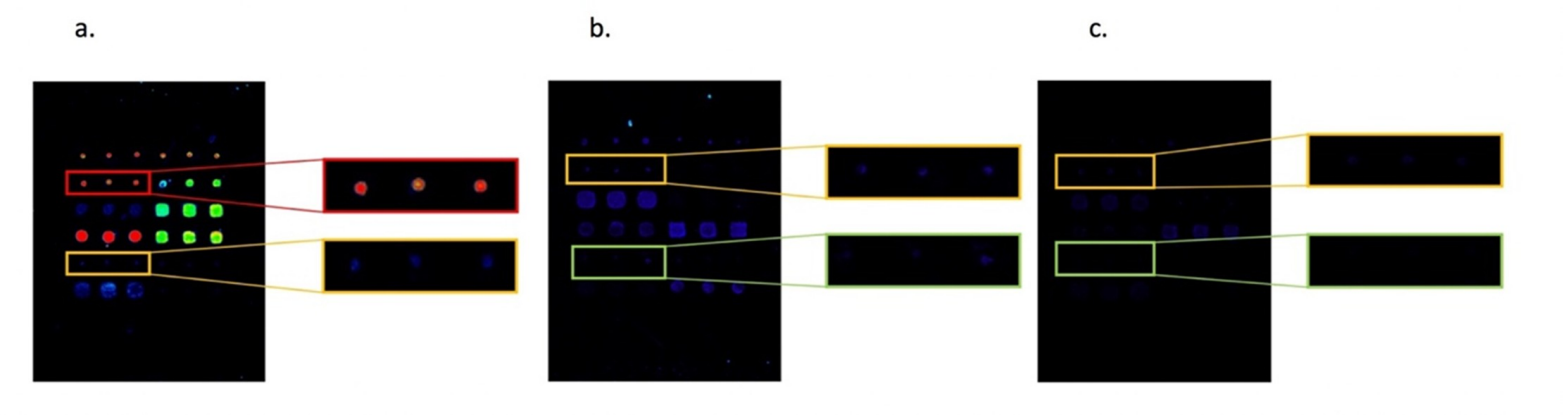
Graphic representation of the fluorescence intensities obtained from (a) an ACPA-positive RA serum, (b) an ACPA-negative control serum and (c) an ACPA-negative PsA serum.

Our results indicate that these new chimeric citrullinated peptides derived from human proteins should be considered for inclusion in the design of a multiplex citrullinated peptide-based array for the diagnosis of RA. In order to identify the best conditions for performing the multiplex assays, optimization experiments were conducted as described in the Materials and methods section above. We analyzed the diagnostic value of a multiplex array containing the eight different citrullinated chimeric peptides and their arginine counterparts (to control for citrulline specificity), which had previously been validated using another panel of RA sera and the CFFCP antigens [11]. A subset of 70 healthy BDs from the cohort used for ELISA analysis was used to establish the cut-off for each of the chimeric peptide antigens tested. To control for specificity, we used a panel of 70 sera derived from patients affected by PsA, an inflammatory disease with a clinical presentation that can simulate that of RA. First of all, the intra-assay CV was calculated for all sample/peptide pairs. We clearly observed higher fluorescence intensity response units (RUs) for the pairs of RA/citrullinated peptides than for the other pairs studied (BD or PsA/citrullinated peptides or arginine control peptides, and RA/arginine control peptides). In agreement with this, low-level RU samples (BD and PsA) showed overall a higher CV than high-level RU samples (RA). We therefore decided to exclude from the study those sera where CV > 30% in low-level RU and those with CV > 25% in samples with high intensity (Table A in S1 File). At least two spots in a triplicate had to be accepted to obtain results. Several examples of a positive RA serum and negative sera (PsA and BD) are shown in Fig 3.

The association between the measurements performed by microarray and ELISA was made using uncorrected values. Comparison of the fluorescence intensities with the corresponding optical density units yielded satisfactory Spearman’s rank correlation coefficients (ρ_S_): values between 0.70 and 0.83 (Fig D in S1 File). Moreover, the performance of each individual chimeric peptide in the multiplex system was compared with its performance in the ELISA assay. As shown in the ROC curves obtained (Fig E in S1 File), at a level of specificity of 98%, the individual chimeric peptides showed marked differences in sensitivity which also depended on whether the arginine control peptides were subtracted. In general, this correction improved the sensitivity of the corresponding assay (increased the number of RA subjects that were positive), with the only exception of CFVCP, where the number of positive sera decreased (sensitivity values decreased from 36.8% to 30.0%). For CFECP and CEFCP, the effect of subtraction of citrulline control reactivity was null and did not affect the corresponding sensitivity values (Table 1). So, we decided to work with corrected values to measure the sensitivity of the eight chimeric citrullinated peptides in the multiplex array.

**Table 1:** Performance of the eight citrullinated peptides studied and their comparison with the commercial ELISA-based CCP3 test.

Peptide	% of RA sera reacting at 98.0% specificity		% of the same RA sera reacting to CCP3		p
**CFFCP1**	20/34	(58.8%)	30/34	(88.2%)	0.006*
**CFFCP2**	23/34	(67.7%)	31/34	(91.2%)	0.016*
**CFFCP3**	35/44	(79.6%)	38/44	(86.4%)	0.395
**CFECP**	30/38	(79.0%)	33/38	(86.8%)	0.361
**CFVCP**	21/57	(36.8%)	49/57	(86.0%)	0.000*
**CVECP**	39/46	(84.8%)	39/46	(84.8%)	1.000
**CEFCP**	38/45	(84.4%)	40/45	(88.9%)	0.535
**CVFCP**	42/52	(80.8%)	44/52	(84.6%)	0.604

p: two-sample test of proportions p-value

* p < 0.05

**Fig 4:**
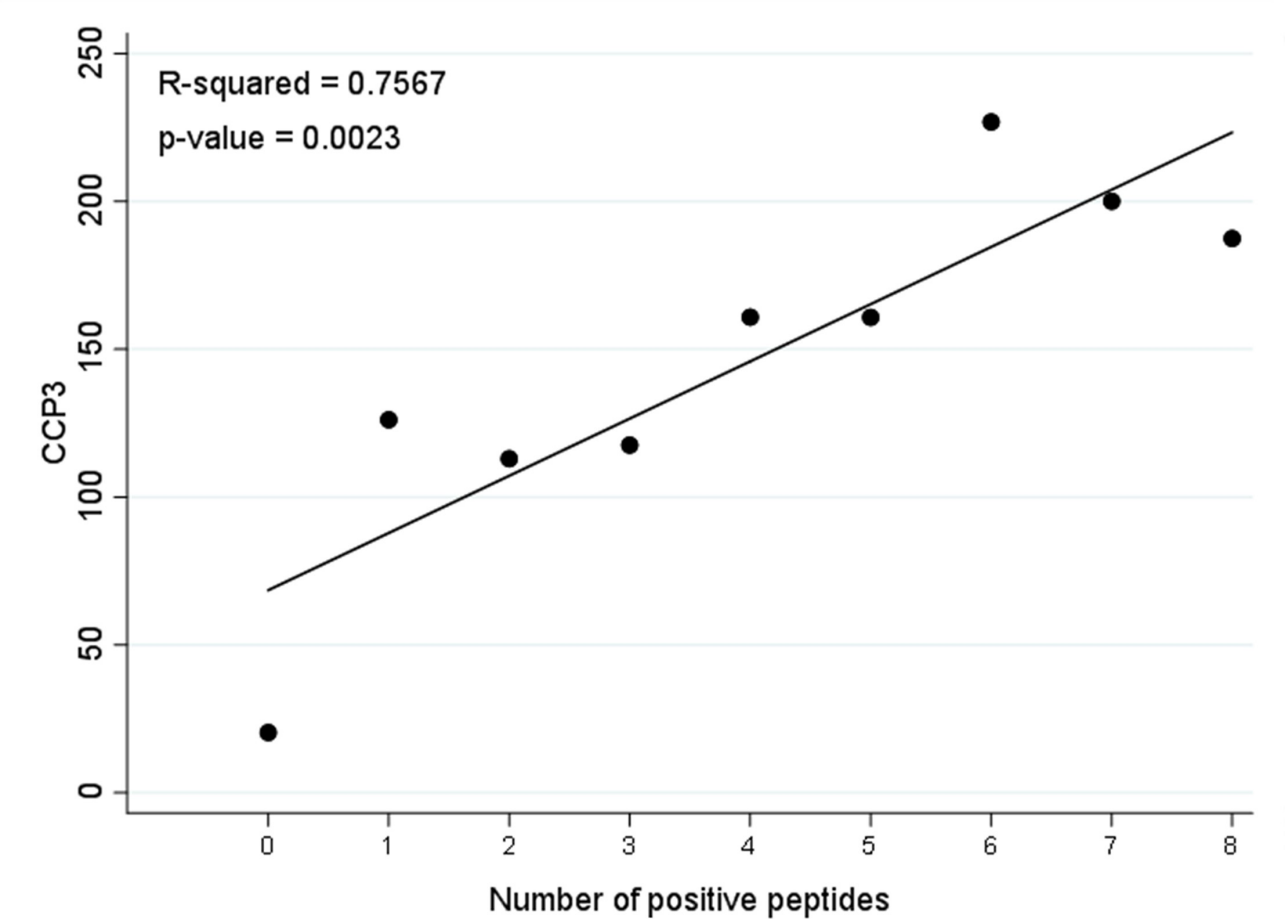
Association between mean CCP3 values and the number of positive citrullinated peptides in the multiplex system. Results of the fitted linear regression model.

Table B in S1 File shows the individual reactivity of each citrullinated chimeric peptide for the RA, healthy BD and PsA cohorts. The results indicate that PsA patients and BD controls behave similarly, and clearly demonstrate very low reactivity compared to RA patients. To compare results of the combination of the eight chimeric citrullinated peptides (patterns) and the CCP3 commercial test for the RA cohort, a linear regression model was fitted (Fig 4).

The model was statistically significant (p = 0.0025) and the coefficient of determination (R-squared) indicates that 75.67% of the variability in CCP3 values is explained by the number of positive citrullinated peptides. Table 1 shows the individual performance of the eight chimeric citrullinated peptides included in the study, in comparison with the commercial CCP3 test, in the RA cohort. As shown in Table 2, three of these peptide sequences behaved significantly discordantly with the commercial test (CFFCP1, CFFPCP2 and CFVCP).

**Table 2:** Sensitivity and specificity analysis of the multiplex assay compared to the commercial ELISA-based CCP3 test, regarding peptide biomarker selection.

**Multiplex: all peptide biomarkers (8)**							
	2+				**3+**		
CCP3	-	+		**CCP3**	**-**	**+**	
-	7	4	11	**-**	**10**	**1**	**11**
+	8	47	55	**+**	**13**	**42**	**55**
	15	51	66		**23**	**43**	**66**
Sensitivity		85,50%		**Sensitivity**		**76,40%**	
Specificity		63,60%		**Specificity**		**90,90%**	
**Multiplex: 5 peptide biomarkers**^**a**^							
	2+				3+		
CCP3	-	+		CCP3	-	+	
-	8	3	11	-	10	1	11
+	11	43	54	+	19	35	54
	19	46	65		29	36	65
Sensitivity		79,60%		Sensitivity		64,80%	
Specificity		72,70%		Specificity		90,90%	
**Multiplex: 6 peptide biomarkers**^**b**^							
	2+				3+		
CCP3	-	+		CCP3	-	+	
-	8	3	11	-	10	1	11
+	10	44	54	+	15	39	54
	18	47	65		25	40	65
Sensitivity		81,50%		Sensitivity		72,20%	
Specificity		72,70%		Specificity		90,90%	

CCP3: < 20 = —vs ≥ 20 = +

^a^ CFFCP1, CFFCP2 and CFVCP excluded

^b^ CFFCP1 and CFFCP2 excluded.

Consequently, next we analyzed how the multiplex system works in comparison to CCP3, with regard to the peptides we considered for inclusion in the array. Firstly, the analysis was carried with all the (eight) peptides and considering multiplex positive to be when three or more peptides were positive. In this case, the following values were obtained: 76.4% sensitivity, 90.9% specificity. When the same analysis conditions were used, but excluding CFFCP1, CFFPCP2 and CFVCP (five peptides were included in the analysis), the results were: 64.8% sensitivity, 90.9% specificity. Then, taking into account that CFFCP1 and CFFCP2 behave identically and although the pattern of citrullination in the primary structure of the three CFFCPs is different, they are composed of the same domains as filaggrin and fibrin proteins, we decided to perform the analysis excluding CFFCP1 and CFFCP2 and maintaining CFVCP. In this last case, the multiplex array contained six peptides and we considered it to be positive when three or more peptides were positive, the corresponding values of sensitivity and specificity were: 72.2% and 90.9%, respectively. Finally and similarly to what is previously reported in the literature [13, 14] when array positivity is defined as being positive for more than two or more than one peptide, results were poorly RA-specific. This confirms the importance of critical selection of citrullinated peptides. In view of all these results, summarized in Table 2, we performed the subsequent analysis using the whole panel of eight peptides, as shown in Fig 1.

**Fig 5:**
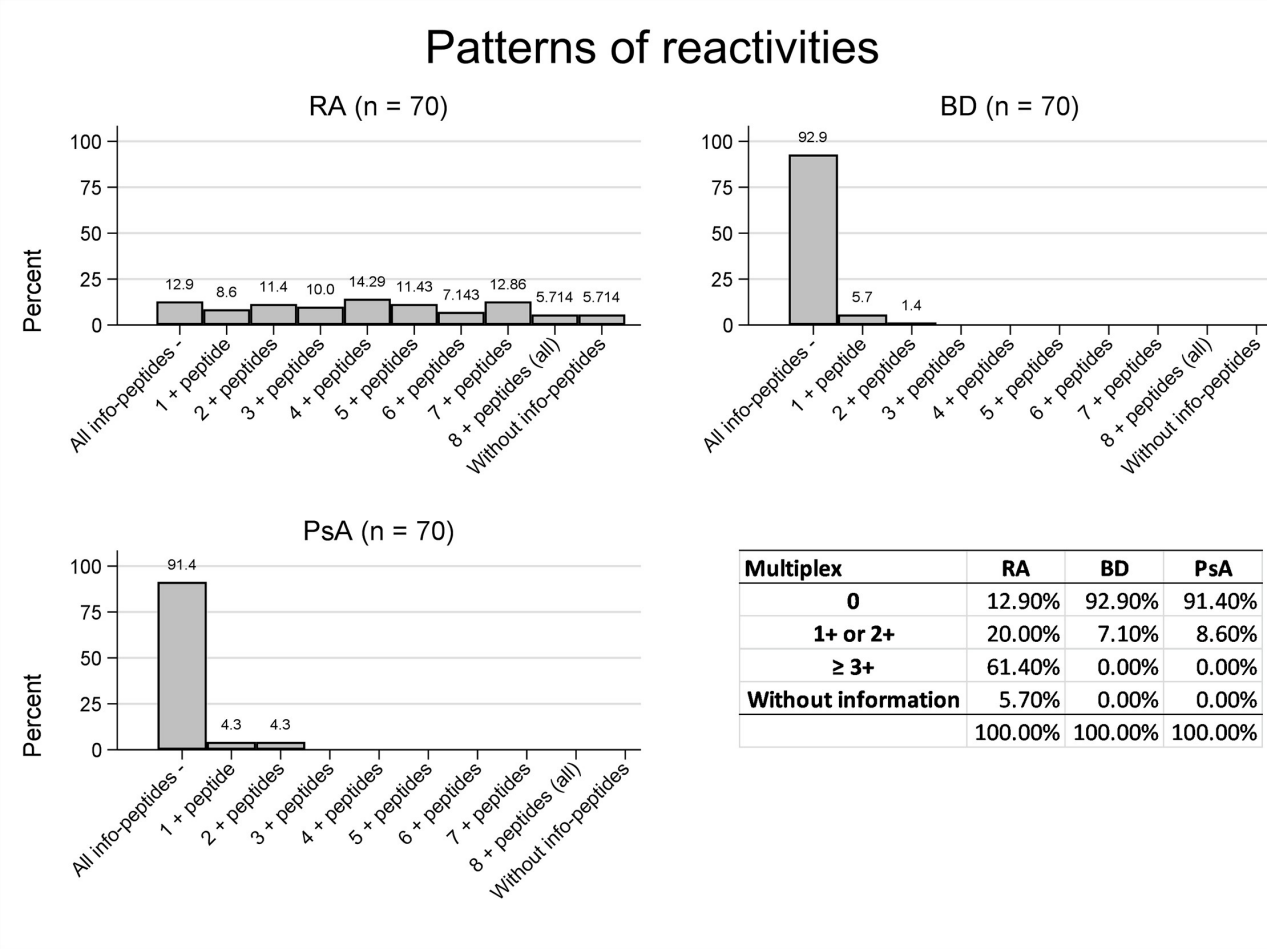
Patterns of reactivities of the eight citrulline peptides in RA patients, healthy BDs and PsA patients.

Fig 5 shows the relative frequency of ACPA positives in the three cohorts (RA and PsA patients; and healthy BD) for the combination of the eight chimeric peptide antigens. As shown, the proportion of RA patients that were positive to 3 or more peptides was 61.4%; while the healthy BD and PsA cohorts showed 0% of these 3-fold ACPA positives.

When array positivity was defined as more than one chimeric peptide antigen being positive, positivity in the RA cohort was 81.4%; while in the two control cohorts, it was 7.1% and 8.6% for BDs and PsA patients respectively. These results demonstrate sensitivity values comparable to those reported by others [15, 16]; while we observed 100% specificity for RA diagnosis when positivity was considered with 3 or more peptides being positive. When comparing with the commercial CCP3 test, discordant results were obtained in 21% of the RA sera studied (13 of 66 were CCP3 positive and multiplex negative; and 1 of 66 resulted CCP3 negative and multiplex positive). These findings reflect the differences that exist in the peptide repertoire in the two platforms and reinforce both the broad heterogeneity of ACPAs and the increasing number of citrullinated proteins or peptides that present different reactivity patterns with RA sera [17]. Moreover, a great number of studies have reported non-specific responses of commercial RA tests [18–20] due to the fact that they never routinely perform a parallel control with the peptides containing native arginine, instead of citrulline. However, our multiplex array easily allows us to perform this control and thus the results reported here for ACPA reactivity are strictly citrulline specific.

## Conclusions

Here, we have develop a novel multiplex platform composed of eight chimeric citrullinated peptides derived from human proteins that abound in rheumatoid synovial, and we have evaluated its suitability to detect anti-citrullinated peptide/protein antibodies (ACPAs) in serum samples of rheumatoid arthritis (RA) and psoriatic arthritis (PsA) patients, as well as in healthy blood donors. Our results demonstrate that the platform has a high specificity for ACPA detection, especially taking into account that the control group consisted of patients affected by PsA, an inflammatory disease whose clinical presentation can simulate that of RA. When comparing with the commercial ELISA-based test (CCP3), it is true that ACPA reactivity was only detected by the multiplex array in one CCP3-negative RA serum sample. However, this in-house multiplex system allowed us to control for background reactivity to arginine and worked in a serum-saving way, compared to CCP3. In conclusion, a highly citrulline-specific multiplex assay based on chimeric citrullinated peptides derived from filaggrin, fibrin, vimentin and human enolase proteins for the detection of ACPAs has been developed herein. In order to improve sensitivity and expand future use in RA diagnosis, peptides bearing other non-proteinogenic amino acids could be designed, synthesized and incorporated into the platform as novel antigenic substrates for further development of the multiplex system.

## Supporting information

S1 File UPLC-MS characterization of CFECP (Fig A), CEFCP (Fig B) and CVECP (Fig C). Relationship between fluorescence intensities (microarray) (RU) and the corresponding optical density units (ELISA) (OD) for RA patients (Fig D). ROC curves analysis from microarray results (Fig E). Number of discarded sera in RA, BD and PsA cohorts (Table A). Reactivity of RA, BD and PsA cohorts to each citrullinated peptide (Table B).
(DOCX)
