# Supplementary material for: Development of a multiplex assay based on chimeric citrullinated peptides as proof of concept for diagnosis of rheumatoid arthritis
Source: PLoS One. 2019 May 2;14(5):e0215927. doi: 10.1371/journal.pone.0215927 (PMC6497438; doi:10.1371/journal.pone.0215927)
Supplement: S1 File — UPLC-MS characterization of CFECP (Fig A), CEFCP (Fig B) and CVECP (Fig C). Relationship between fluorescence intensities (microarray) (RU) and the corresponding optical density units (ELISA) (OD) for RA patients (Fig D). ROC curves analysis from microarray results (Fig E). Number of discarded sera in RA, BD and PsA cohorts (Table A). Reactivity of RA, BD and PsA cohorts to each citrullinated peptide (Table B). (DOCX) [file pone.0215927.s001.docx]

**SUPPLEMENTARY MATERIAL**

**A MULTIPLEX ASSAY BASED ON CHIMERIC CITRULLINATED PEPTIDES FOR THE DIAGNOSIS OF RHEUMATOID ARTHRITIS**

Cristina García-Moreno, María José Gómara, María José Bleda, Raimon Sanmartí and Isabel Haro

**a**


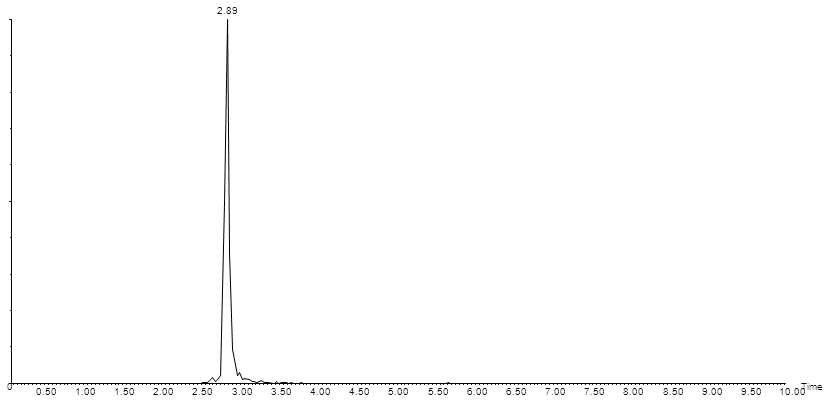


**b**


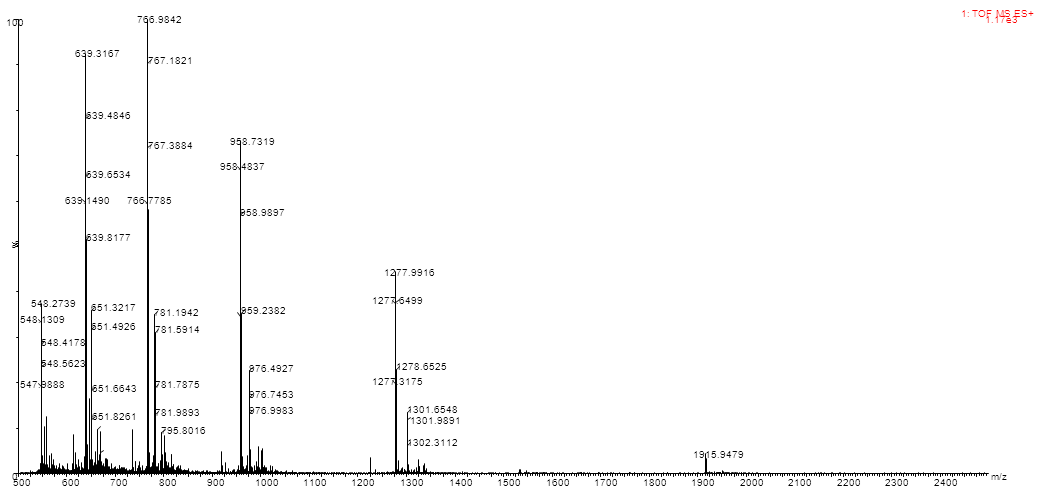


**Figure A**. UPLC-MS characterization of CFECP. (a) Peptide Elution was performed on an Acquity UPLC BEH C18 column (2.1×100 mm, 1.7 μm) with a linear gradient of 5%-100% solvent B (20mM formic acid in ACN) into solvent A (20mM formic acid in water) over 10min at 0.3mL/min. (b) The mass spectrum was recorded in positive ion mode in the m/z 500-2500 range. Calculated m/z: [M+2H]^+2^=1915.46, [M+3H]^+3^=1277.31, [M+4H]^+4^=958.24, [M+5H]^+5^=766.79, [M+6H]^+6^=639.16, [M+7H]^+7^=547.99; experimental m/z: [M+2H]^+2^=1915.95, [M+3H]^+3^=1277.31, [M+4H]^+4^=958.48, [M+5H]^+5^=766.77, [M+6H]^+6^=639.15, [M+7H]^+7^=547.99

**a**


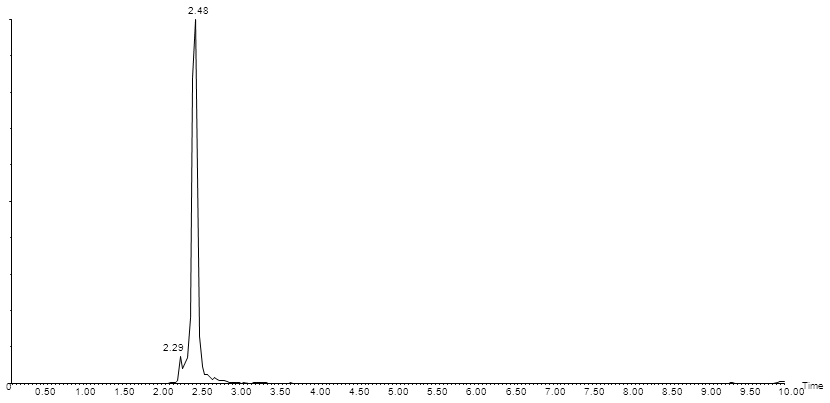


**b**


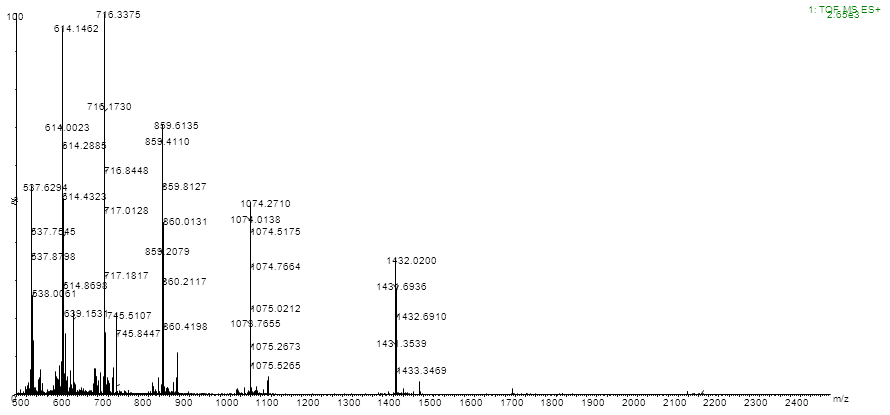


**Figure B**. UPLC-MS characterization of CEFCP. (a) Peptide Elution was performed on an Acquity UPLC BEH C18 column (2.1×100 mm, 1.7 μm) with a linear gradient of 5%-100% solvent B (20mM formic acid in ACN) into solvent A (20mM formic acid in water) over 10min at 0.3mL/min. (b) The mass spectrum was recorded in positive ion mode in the m/z 500-2500 range. Calculated m/z: [M+3H]^+3^=1431.02, [M+4H]^+4^=1073.51, [M+5H]^+5^=859.01; [M+6H]^+6^=716.01, [M+7H]^+7^=613.87, [M+8H]^+8^=537.26; experimental m/z: [M+3H]^+3^=1431.35, [M+4H]^+4^=1073.76, [M+5H]^+5^=859.20; [M+6H]^+6^=716.17, [M+7H]^+7^=614.00, [M+8H]^+8^=537.62

**a**


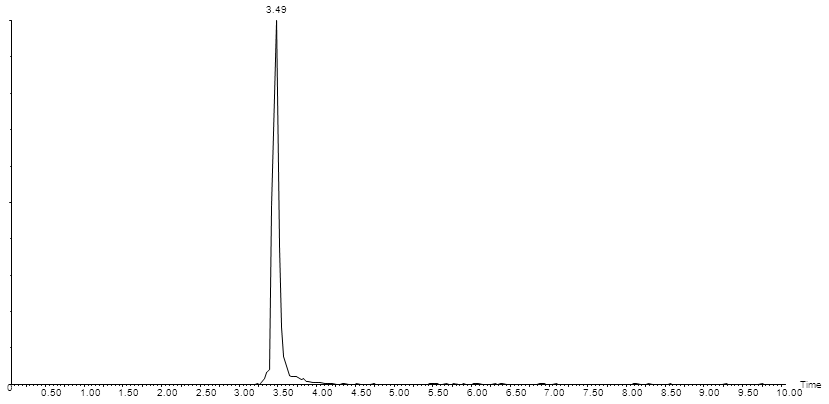


**b**


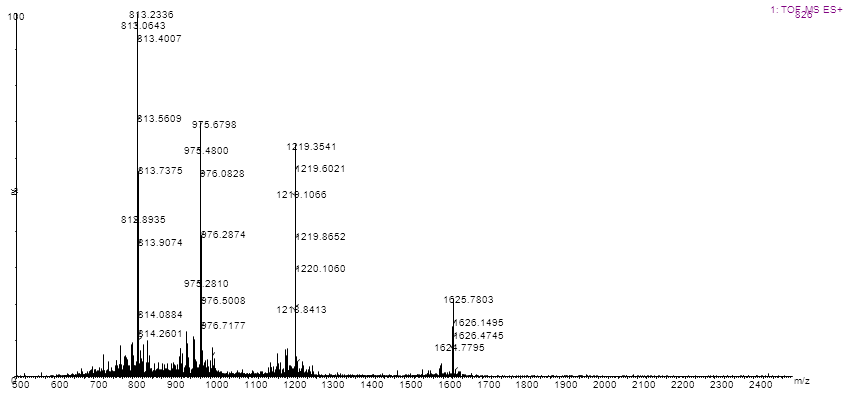


**Figure C**. UPLC-MS characterization of CVECP. (a) Peptide Elution was performed on an Acquity UPLC BEH C18 column (2.1×100 mm, 1.7 μm) with a linear gradient of 5%-100% solvent B (20mM formic acid in ACN) into solvent A (20mM formic acid in water) over 10min at 0.3mL/min. (b) The mass spectrum was recorded in positive ion mode in the m/z 500-2500 range. Calculated m/z: [M+3H]^+3^=1625.14, [M+4H]^+4^=1219.11, [M+5H]^+5^=975.49; [M+6H]^+6^=813.07; experimental m/z: [M+3H]^+3^=1624.78, [M+4H]^+4^=1218.84, [M+5H]^+5^=975.28; [M+6H]^+6^=812.89


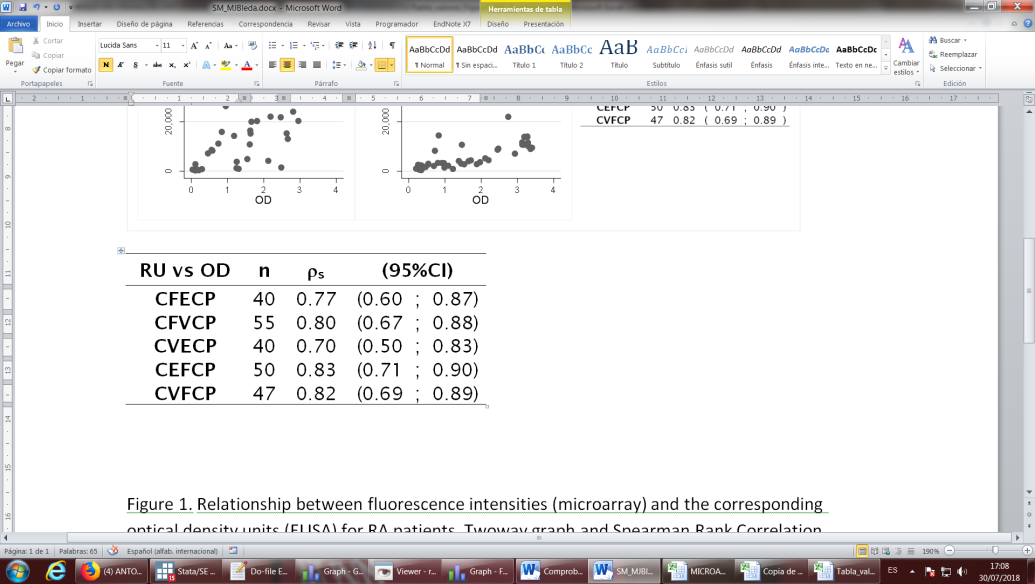


**Figure D**. Relationship between fluorescence intensities (microarray) (RU) and the corresponding optical density units (ELISA) (OD) for RA patients (n). Two-way graphs and Spearman’s rank correlation coefficients (ρ_s_) with their 95% confidence interval (95%CI).

**Figure E**. ROC curves analysis from microarray results with chimeric fibrin-filaggrin citrullinated peptides (CFFCP1, CFFCP2, CFFCP3), chimeric vimentin-filaggrin citrullinated peptide (CVFCP), chimeric fibrin-vimentin citrullinated peptide (CFVCP), chimeric enolase-filaggrin citrullinated peptide (CEFCP), chimeric vimentin-enolase citrullinated peptide (CVECP) and chimeric fibrin-enolase citrullinated peptide (CFECP) in the RA and BD cohorts of patients.

**Table A**. Number of discarded sera in RA, BD and PsA cohorts due to a high variability in the fluorescence intensity response units of corresponding triplicates spots for citrullinated and control peptides.

| **RA (n = 70)** | | | | | | | | |
| --- | --- | --- | --- | --- | --- | --- | --- | --- |
|  | **Discarded sera**  **CV > 25%** | | **Control peptide** | **Discarded sera**  **CV > 30%** | | **Discarded pairs**  **Peptide or Control CV > 25%,30%** | | **Final pairs** |
| **CFFCP1** | **20** | (28.57%) | **CFFCP-R** | **21** | (30.00%) | **36** | (51.43%) | **34** |
| **CFFCP2** | **21** | (30.00%) | **CFFCP-R** | **21** | (30.00%) | **36** | (51.43%) | **34** |
| **CFFCP3** | **10** | (14.29%) | **CFFCP-R** | **21** | (30.00%) | **26** | (37.14%) | **44** |
| **CFECP** | **16** | (22.86%) | **CFECP-R** | **21** | (30.00%) | **32** | (45.71%) | **38** |
| **CFVCP** | **0** | (0.00%) | **CFVCP-R** | **13** | (18.57%) | **13** | (18.57%) | **57** |
| **CVECP** | **15** | (21.43%) | **CVECP-R** | **13** | (18.57%) | **24** | (34.29%) | **46** |
| **CEFCP** | **6** | (8.57%) | **CEFCP-R** | **21** | (30.00%) | **25** | (35.71%) | **45** |
| **CVFCP** | **8** | (11.43%) | **CVFCP-R** | **13** | (18.57%) | **18** | (25.71%) | **52** |
| **BD (n = 70)** | | | | | | | | |
|  | **Discarded sera**  **CV > 30%** | | **Control peptide** | **Discarded sera**  **CV > 30%** | | **Discarded pairs**  **Peptide or Control CV > 30%** | | **Final pairs** |
| **CFFCP1** | **7** | (10.00%) | **CFFCP-R** | **14** | (20.00%) | **20** | (28.57%) | **50** |
| **CFFCP2** | **20** | (28.57%) | **CFFCP-R** | **14** | (20.00%) | **30** | (42.86%) | **40** |
| **CFFCP3** | **14** | (20.00%) | **CFFCP-R** | **14** | (20.00%) | **24** | (34.29%) | **46** |
| **CFECP** | **10** | (14.29%) | **CFECP-R** | **11** | (15.71%) | **18** | (25.71%) | **52** |
| **CFVCP** | **3** | (4.29%) | **CFVCP-R** | **7** | (10.00%) | **10** | (14.29%) | **60** |
| **CVECP** | **13** | (18.57%) | **CVECP-R** | **7** | (10.00%) | **17** | (24.29%) | **53** |
| **CEFCP** | **3** | (4.29%) | **CEFCP-R** | **11** | (15.71%) | **13** | (18.57%) | **57** |
| **CVFCP** | **8** | (11.43%) | **CVFCP-R** | **7** | (10.00%) | **14** | (20.00%) | **56** |
| **PsA (n = 70)** | | | | | | | | |
|  | **Discarded sera**  **CV > 30%** | | **Control peptide** | **Discarded sera**  **CV > 30%** | | **Discarded pairs**  **Peptide or Control CV > 30%** | | **Final pairs** |
| **CFFCP1** | **9** | (12.86%) | **CFFCP-R** | **3** | (4.29%) | **12** | (17.14%) | **58** |
| **CFFCP2** | **29** | (41.43%) | **CFFCP-R** | **3** | (4.29%) | **32** | (45.71%) | **38** |
| **CFFCP3** | **11** | (15.71%) | **CFFCP-R** | **3** | (4.29%) | **14** | (20.00%) | **56** |
| **CFECP** | **8** | (11.43%) | **CFECP-R** | **14** | (20.00%) | **18** | (25.71%) | **52** |
| **CFVCP** | **1** | (1.43%) | **CFVCP-R** | **6** | (8.57%) | **7** | (10.00%) | **63** |
| **CVECP** | **10** | (14.29%) | **CVECP-R** | **6** | (8.57%) | **15** | (21.43%) | **55** |
| **CEFCP** | **3** | (4.29%) | **CEFCP-R** | **14** | (20.00%) | **17** | (24.29%) | **53** |
| **CVFCP** | **7** | (10.00%) | **CVFCP-R** | **6** | (8.57%) | **13** | (18.57%) | **57** |

**Table B**. Reactivity of RA, BD and PsA cohorts to each citrullinated peptide.

|  | **RA** | | | **BD** | | | **PsA** | | |
| --- | --- | --- | --- | --- | --- | --- | --- | --- | --- |
|  | Number of sera reacting | **Number of positive** | (%) | Number of sera reacting | **Number of positive** | (%) | Number of sera reacting | **Number of positive** | (%) |
| **CFFCP1** | 34 | **20** | (58.8) | 50 | **1** | (2.0) | 58 | **0** | (0.0) |
| **CFFCP2** | 34 | **23** | (67.7) | 40 | **0** | (0.0) | 38 | **1** | (2.6) |
| **CFFCP3** | 44 | **35** | (79.6) | 46 | **0** | (0.0) | 56 | **0** | (0.0) |
| **CFECP** | 38 | **30** | (80.0) | 52 | **1** | (1.9) | 52 | **5** | (9.6) |
| **CFVCP** | 57 | **21** | (36.8) | 60 | **1** | (1.7) | 63 | **0** | (0.0) |
| **CVECP** | 46 | **39** | (84.8) | 53 | **1** | (1.9) | 55 | **3** | (5.5) |
| **CEFCP** | 45 | **38** | (84.4) | 57 | **1** | (1.8) | 53 | **0** | (0.0) |
| **CVFCP** | 52 | **42** | (80.8) | 56 | **1** | (1.8) | 57 | **0** | (0.0) |
